# Factors associated with population coverage of targeted malaria elimination (TME) in southern Savannakhet Province, Lao PDR

**DOI:** 10.1186/s12936-017-2070-y

**Published:** 2017-10-23

**Authors:** Bipin Adhikari, Koukeo Phommasone, Tiengkham Pongvongsa, Palingnaphone Kommarasy, Xayaphone Soundala, Gisela Henriques, Nicholas J. White, Nicholas P. J. Day, Arjen M. Dondorp, Lorenz von Seidlein, Phaik Yeong Cheah, Christopher Pell, Mayfong Mayxay

**Affiliations:** 1 Mahidol-Oxford Tropical Medicine Research Unit, Faculty of Tropical Medicine, Mahidol University, Bangkok, Thailand; 2 Centre for Tropical Medicine and Global Health, Nuffield Department of Medicine, Churchill Hospital, Oxford, UK; 3 Kellogg College, University of Oxford, Oxford, UK; 4 Lao-Oxford-Mahosot Hospital-Wellcome Trust Research Unit (LOMWRU), Microbiology Laboratory, Vientiane, Laos; 5 Savannakhet Provincial Health Department, Savannakhet, Savannakhet Province Laos; 6 The Ethox Centre, Nuffield Department of Population Health, University of Oxford, Oxford, UK; 7 Centre for Social Science and Global Health, University of Amsterdam, Amsterdam, The Netherlands; 8 Amsterdam Institute for Global Health and Development, Amsterdam, The Netherlands; 9 Faculty of Postgraduate Studies, University of Health Sciences, Vientiane, Laos

**Keywords:** Malaria, Elimination, Community, Engagement, Acceptability, Knowledge, Trust

## Abstract

**Background:**

Targeted malaria elimination (TME) in Lao PDR (Laos) included three rounds of mass drug administrations (MDA) against malaria followed by quarterly blood surveys in two villages in Nong District at Savannakhet Province. The success of MDA largely depends upon the efficacy of the anti-malarial drug regimen, local malaria epidemiology and the population coverage. In order to explore the reasons for participation in TME, a quantitative survey was conducted after the completion of the three rounds of MDA.

**Methods:**

The survey was conducted in two villages with a total of 158 households in July and August 2016. Among the 973 villagers eligible for participation in the MDA, 158 (16.2%) adults (> 18 years) were selected, one each from every household for the interviews using a quantitative questionnaire.

**Results:**

150/158 (94.9%) respondents participated at least in one activity (taking medicine or testing their blood) of TME. 141/150 (94.0%) respondents took part in the MDA and tested their blood in all three rounds. 17/158 (10.7%) were partial or non-participants in three rounds of MDA. Characteristics of respondents which were independently associated with completion of three rounds of MDA included: attending TME meetings [AOR = 12.0 (95% CI 1.1–20.5) (p = 0.03)], knowing that malaria can be diagnosed through blood tests [AOR = 5.6 (95% CI 1.0–32.3) (p = 0.05)], all members from household participated [AOR = 4.2 (95% CI 1.3–14.0) (p = 0.02)], liking all aspects of TME [AOR = 17.2 (95% CI 1.6–177.9) (p = 0.02)] and the perception that TME was important [AOR = 14.9 (95% CI 1.3–171.2) (p = 0.03)].

**Conclusion:**

Complete participation in TME was significantly associated with participation in community engagement activities, knowledge that the blood tests were for malaria diagnosis, family members’ participation at TME and perceptions that TME was worthwhile. A responsive approach to community engagement that includes formative research and the involvement of community members may increase the uptake of the intervention.

**Electronic supplementary material:**

The online version of this article (doi:10.1186/s12936-017-2070-y) contains supplementary material, which is available to authorized users.

## Background

**Fig. 1:**
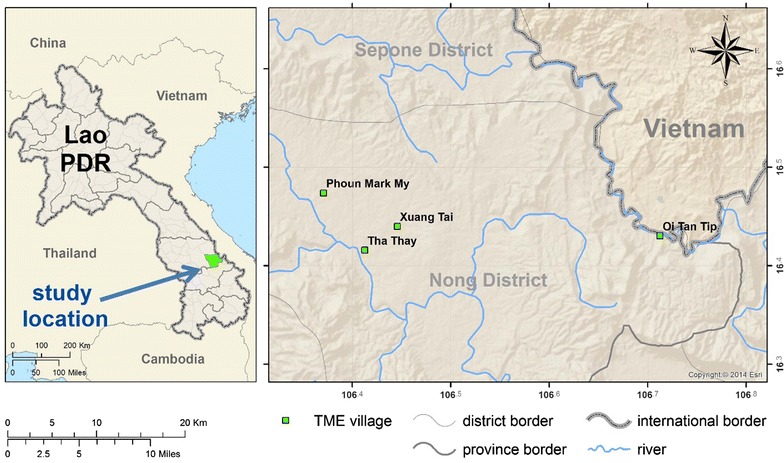
TME study sites in Savannakhet Province of Laos

The spread of multidrug resistant *Plasmodium falciparum* in the Greater Mekong sub-Region has added urgency to malaria elimination efforts [1–5]. Targeted malaria elimination (TME) has been proposed as a multi-pronged strategy to accelerate elimination in the region. The approach comprises: (1) the strengthening of village malaria workers (VMWs) to provide appropriate case management and distribute long-lasting insecticide-treated bed nets (LLINs) and (2) mass drug (anti-malarial) administration (MDA) and quarterly blood survey (Fig. 1). To date, this strategy is being evaluated in the Thai–Myanmar border area, Cambodia, Vietnam and Laos [6].

The pilot TME studies aim to interrupt local *falciparum* malaria transmission [6]. The probability of accomplishing this through MDA depends on the dynamics of local malaria transmission, the efficacy of the anti-malarial regimen and coverage in the target populations [7]. Achieving a sufficiently high uptake in the target population—estimated at around 80% of all residents—is challenging for several reasons [7, 8]. For example, target communities in the Greater Mekong sub-region, where malaria transmission persists, are often isolated with limited healthcare infrastructure; apparently healthy, participants must adhere to the complete treatment regimen [9]; and concerns about potential and real side effects can discourage uptake and adherence [10].

To maximize coverage in target populations, community engagement often accompanies MDA [6–8]. This entails a range of activities to support and facilitate the uptake of an intervention and adherence, such as providing health education during community meetings or house-to-house visits [7, 11]. Community engagement is also a means of promoting sustainable change through increasing the health literacy and building local capacity [11–13].

To date, several questionnaire-based studies have examined the factors that influence coverage of mass anti-malarial administration [9, 14]. These studies found that investments in providing information to villagers through trustworthy informants were essential to increase participation. No research has so far focused on the uptake of MDA in Laos, where this strategy has also been evaluated. In light of the specific social, cultural, health system and epidemiological circumstances in Laos, with a view to informing current and future malaria elimination campaigns, this article explores the factors associated with participation in MDA as a part of TME.

## Methods

### Intervention villages

**Fig. 2:**
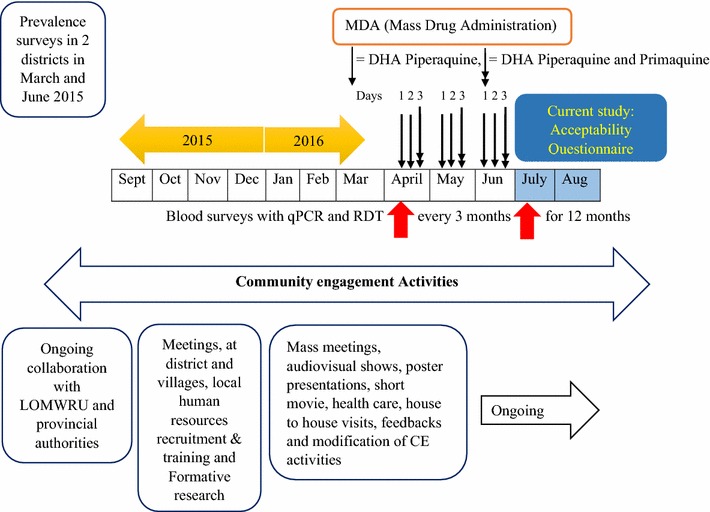
Schematic diagram of MDA, CE, blood survey and acceptability questionnaire interviews

In 2016, MDA took place in two TME intervention villages (PhounMakMee: PMM; and Thathay: TT), located in remote Nong District, Savannakhet Province close to the Vietnam border (Fig. 1). These villages were selected according to a 2015 malaria prevalence survey, which was conducted in two districts of Savannakhet Province [15]. Villagers were given anti-malarials as directly-observed therapy (DOT). The anti-malarial regimen consisted of three rounds of 3 days of dihydroartemisinin piperaquine (DHAP) and a single low dose of primaquine (PQ) at monthly intervals (Fig. 2). Blood samples were collected before the mass antimalarial administration and then every 3 month for 12 months to detect and quantify parasitaemia [6].

The residents of the intervention villages are mostly (96.8%; 153/158) from the *Lao Theung* ethnic group, who are Mon-Khamer speaking aboriginals whose oral language is incomprehensible to the majority (*Lao Lum*) ethnic group in Laos (Table 1). About one-third of villagers are literate and the majority (90.5%) attended less than 5 years of school education. The majority (93%) of villagers are farmers and practice swidden cultivation of staple foods, mainly rice. Income generation is mostly based on rearing of domestic animals such as pigs, cows, buffaloes, chicken and goats, which are also a source of emergency cash [16].

**Table 1:** Socio-demographic and economic characteristics of the respondents in relation to participation (n = 158)

Characteristics	Participation			p value
	Partial/none (n = 17)	Complete (n = 141)	Total (n = 158)	
	Number (%)	Number (%)	Number (%)	
Respondent status				
Family head	12 (70.6)	88 (62.4)	100 (63.3)	0.35
Other	5 (29.4)	53 (37.6)	58 (36.7)	
Age group (years)				
≤ 29	6 (35.3)	47 (33.3)	53 (33.5)	0.72
30–40	7 (41.2)	48 (34)	55 (34.8)	
≥ 41	4 (23.5)	46 (32.6)	50 (31.6)	
Sex				
Female	5 (29.4)	29 (20.6)	34 (21.5)	0.28
Male	12 (70.6)	112 (79.4)	124 (78.5)	
Ethnicity				
Lao Lum	1 (5.9)	1 (0.7)	2 (1.3)	0.16
Lao Theung	16 (94.1)	137 (97.2)	153 (96.8)	
Other	0	3 (2.1)	3 (1.9)	
Religion				
Animist	16 (94.1)	138 (97.9)	154 (97.5)	0.36
Buddhist	1 (5.9)	3 (2.1)	4 (2.5)	
Marital status				
In relationship	17 (100)	129 (91.5)	146 (92.4)	0.24
Not in relationship	0	12 (8.5)	12 (7.6)	
Literacy				
Illiterate	14 (82.4)	101 (71.6)	115 (72.8)	0.26
Literate	3 (17.6)	40 (28.4)	43 (27.2)	
Education in years				
≤ 5	16 (94.1)	127 (90.1)	143 (90.5)	0.5
≥ 5.1	1 (5.9)	14 (9.9)	15 (9.5)	
Occupation				
Farmer	16 (94.1)	131 (92.9)	147 (93)	0.66
Other	1 (5.9)	10 (7.1)	11 (7)	
Monthly income (kip)				
≤ 500,000	16 (94.1)	124 (87.9)	140 (88.6)	0.72
≥ 500,001	1 (5.9)	15 (10.6)	16 (10.1)	
Don’t know	0	2 (1.4)	2 (1.3)	
Presence of toilet facility at home				
Yes	3 (17.6)	18 (12.8)	21 (13.3)	0.4
No	14 (82.4)	123 (87.2)	137 (86.7)	
Migrated from other village				
Yes	6 (35.3)	45 (31.9)	51 (32.3)	0.48
No	11 (64.7)	96 (68.1)	107 (67.7)	
Distance between forest and house in km				
≤ 1	10 (62.5)	82 (59.9)	92 (60.1)	0.53
≥ 1.1	6 (37.5)	55 (40.1)	61 (39.9)	
Frequency of visit to forest				
Everyday	9 (52.9)	87 (61.7)	96 (60.8)	0.77
≥ Every alternate day	7 (41.2)	48 (34)	55 (34.8)	
NA	1 (5.9)	6 (4.3)	7 (4.4)	

Alongside TME in Laos, community engagement comprised five key elements. (1) The study entailed stepwise process that involved meetings with authorities at various levels before initiating village-level activities [16]. (2) Formative research (knowledge, attitudes and perceptions towards malaria and MDA) was conducted to formulate an appropriate approach at village level e.g. designing material that used pictorial explanations for TME because of villagers’ low levels of literacy. (3) With the assistance of local leaders, villagers were selected and trained as volunteers who coordinated village-level meetings to inform villagers about the TME. (4) These meetings were part of the responsive approach whereby volunteers listened to and recorded their concerns so as to be able to adapt subsequent activities, for example, by conducting house-to-house visits, to respond to emerging rumors. (5) Community meetings—and all TME activities at the village level—were jointly decided upon by TME staff and village volunteers. This shared leadership and decision-making [16] is a core element of community-directed interventions and recognized as important to garner villagers’ trust and participation [17, 18].

The health education tools, which were used during mass meetings and one-to-one community engagement, including videos about TME and MDA made by the study team, a malaria guide book with pictorial representation of the concept of TME, and a T-shirt with a message about malaria elimination. The study team made use of these to explain malaria transmission, prevention, treatment and elimination. These activities were intended to improve villagers’ understanding of the MDA, the blood draws and of malaria in general—issues that have been recognized as barriers to participation [19, 20].

### Data collection

To assess villagers’ socio-demographic characteristics, their knowledge, attitudes, perceptions and experiences regarding TME, a questionnaire-based survey was conducted in July and August 2016 following three rounds of MDA (Additional file 1). All households (n = 158) within the intervention villages were included in the survey. One adult (above 18 years) from each house was interviewed. One of two trained social scientists approached the household head at his/her residence and asked his/her consent to participate in the survey. If the household head was not present, the interviewer sought consent from and interviewed any other adult household member. If consent was given, the questionnaire was administered face-to-face at the respondent’s household. The majority of the questionnaires were administered in *Lao Theung* (127/158; 80.4%) with the assistance of trained local volunteers who could translate between *Pasha Lao* and *Lao Theung*. Each survey lasted about 20–30 min.

The questionnaire was adapted from a version used to assess the same factors in diverse settings, including The Gambia [20], Thai-Myanmar border [9] and Vietnam [14]. The questionnaire was translated, pre-tested and checked for clarity, language and comprehensibility with Laotian researchers at Laos-Oxford Mahosot Wellcome Trust Research Unit in Vientiane, then with 20 respondents in Vientiane, and finally at the Nong District headquarter with local household heads (n = 6). After each round of pre-testing minor revisions were made.

The questionnaire (Additional file 1) contains five sections (Section I: Consent, interviewer’s initials, date, language of interview and participation in TME, Section II: Socio-demographic characteristics of the study participants, Section III: Knowledge about malaria and MDA, Section IV: Experience on TME and Section V: Perceptions on TME). All variables broadly representing these sections were analysed with the outcome variable “participation in TME”.

### Data management and analysis

The questionnaires were single entered into a Microsoft Excel spreadsheet. Consistency and outlying data were cross-checked against the paper questionnaire, which was used to collect data. Participation in MDA was re-categorized into (1) complete participation and (2) none/partial participation. Complete participation referred to respondents who took all nine doses of MDA with DHA Piperaquine and partial or none referred to respondents who took fewer than nine doses or did not participate at all. Initial analysis included frequency and percentage of socio-demographic variables in relation to participation. Comparisons were made using Chi squared test or Fisher exact test as appropriate. Significant associations were considered if p value ≤ 0.05. For logistic regression, all significant predictor including outcome variable were recoded into dichotomy, “0” representing “absence or no” and “1” representing “presence or yes”. Considering the high correlation of the variables under a similar theme, variables representing a question or a theme relevant to research question were selected for univariate and multivariate analysis. A logistic regression model was used to test the association between the predicting variables and the outcome variables (0 = partial/none participation and 1 = complete participation). Variables, thematically relevant to research question, such as participation in meetings, knowledge about MDA, experience of participating in MDA and perceptions towards MDA, were explored and included in the final logistic regression model adjusting the effect of confounders. The fitness of the model was assessed using Omnibus Test of model coefficients (p ≤ 0.05) and Hosmer and Lemeshow Test (with p ≥ 0.05). Data were analysed using IBM SPSS version 24.

### Ethics

Ethical approval for the study was received from the Lao National Ethics Committee for Health Research (Ref. No. 013-2015/NECHR), Government of the Lao PDR and the Oxford Tropical Research Ethics Committee (1015-13).

## Results

### Participation in MDA and TME

The questionnaire was administered in two villages, with a combined population of 1017 (according to the TME census conducted in July 2016). Of these villagers, 973 were eligible for MDA, after excluding infants under 6 months, pregnant women and severely sick people. Of 973 residents, 855 (87.8%) participated in TME (blood survey and three rounds of MDA, based on the preliminary analysis). The questionnaire was administered to 158/1017 (16.2%) adults from 158 households in the intervention villages. Most respondents (150/158; 94.9%) participated in TME with 141 complete participants (141/150; 94%), who took the anti-malarial and had their blood tested in all 3 monthly rounds and seventeen (17/158; 10.7%) partial or non-participants. Among these 17 partial or non-participants, nine (9/17; 52.9%) took part in at least one round of MDA and blood testing, eight (47.1%) did not participate at all (Table 1 and Additional file 2). The complete non-participants, did not take part in MDA and blood test for several reasons including “fear of the blood test”. Nine other respondents, could not complete the participation because s/he “was travelling”, “was busy”, “was pregnant” and “developed adverse events due to the medicine”.

### Socio-demographic characteristics

Most respondents were from the *Lao Theung* ethnic group (153/158; 96.8%) (Table 1). Respondents reported limited education (143/158; 90.5% had < 5 years of education), high illiteracy and low socio-economic status (140/158; 88.6% had monthly income of < 60USD). Only a few (21/158; 13.3%) had access to a latrine at home and most defecated in the forest/fields. None of these socio-demographic characteristics were associated with participation in TME.

### Knowledge about MDA and malaria, experience of and perceptions towards TME

Several factors were associated with complete participation in TME. Respondents who attended TME meetings and had knowledge of malaria symptoms, diagnosis of malaria in TME (through blood test) were more likely to complete all three rounds of MDA (Table 2). Respondents were more likely to complete participation if their household members participated and had fewer complaints (Table 3). Respondents who felt that they have received enough information about TME, and had understood the study rationale and had positive impression about TME were more likely to participate in all rounds of MDA (Table 4).

**Table 2:** Knowledge about TME and Malaria of the respondents in relation to participation (n = 158)

Characteristics	Participation			p value
	Partial/none (n = 17)	Complete (n = 141)	Total (n = 158)	
	Number (%)	Number (%)	Number (%)	
Heard about the current malaria elimination project				
Yes	17 (100)	141 (100)	158 (100)	NA
Heard through/from^a^				
District Health Team/Village Health Workers/Study Staffs	13 (76.5)	137 (97.2)	150 (94.9)	*0.005*
Neighbor	0	1 (0.6)	1 (0.6)	0.89
Village head	10 (58.8)	109 (77.3)	119 (75.3)	0.089
Don’t know	1 (5.9)	4 (2.8)	5 (3.2)	0.43
Attended meetings/events conducted by TME				
Yes	11 (64.7)	138 (97.9)	149 (94.3)	<* 0.001*
No	6 (35.3)	3 (2.1)	9 (5.7)	
TME was explained to you by^a^				
Village head	10 (58.8)	124 (87.9)	134 (84.8)	*0.005*
Volunteers	9 (52.9)	117 (83)	126 (79.7)	*0.008*
TME staffs	9 (52.9)	132 (93.6)	141 (89.2)	<* 0.001*
Frequency of explanation about TME by study staffs				
Up to 30 times	8 (47.1)	128 (90.8)	136 (86.1)	< *0.001*
Can’t remember/don’t know	9 (52.9)	13 (9.2)	22 (13.9)	
Frequency of explanation about TME by non-study staffs				
Up to 20 times	10 (58.8)	137 (97.2)	147 (93)	<* 0.001*
Can’t remember/don’t know	7 (41.2)	4 (2.8)	11 (7)	
We get malaria from^a^				
Forest	2 (11.8)	2 (1.4)	4 (2.5)	0.058
Mosquito	14 (82.4)	139 (98.6)	153 (96.8)	*0.009*
Signs and symptoms of malaria^a^				
Fever	8 (47.1)	115 (81.6)	123 (77.8)	*0.003*
Headache	7 (41.2)	105 (74.5)	112 (70.9)	*0.007*
Muscle pain	1 (5.9)	14 (9.9)	15 (9.5)	0.5
Vomiting	1 (5.9)	7 (5)	8 (5.1)	0.6
Chills/shivering	8 (47.1)	116 (82.3)	124 (78.5)	*0.003*
Diarrhea	1 (5.9)	7 (5)	8 (5.1)	0.6
Don’t know	6 (35.3)	15 (10.6)	21 (13.3)	*0.013*
Diagnosis of malaria^a^				
Through blood test	10 (58.8)	128 (90.8)	138 (87.3)	*0.002*
That person will have fever, chills and headache	0	7 (5)	7 (4.4)	0.44
Went to health worker	14 (82.4)	117 (83)	131 (82.9)	0.58
Went to forest before	2 (11.8)	0	2 (1.3)	*0.011*
An asymptomatic villager can have malaria parasite				
Yes	3 (17.6)	60 (42.6)	63 (39.9)	*0.04*
No	1 (5.9)	19 (13.5)	20 (12.7)	
Don’t know	13 (76.5)	62 (44)	75 (47.5)	
Ways to eliminate malaria from the village^a^				
By giving medicine to all the villagers	6 (35.3)	117 (83)	123 (77.8)	<* 0.001*
By using mosquito nets	1 (5.9)	6 (4.3)	7 (4.4)	0.55
By cleaning the surrounding	0	2 (1.4)	2 (1.3)	0.79
Don’t know	9 (52.9)	18 (12.8)	27 (17.1)	<* 0.001*

^a^Multiple answers were possible; percentage exceeds 100; analysis were made between “Yes” and “No”

**Table 3:** Experiences of TME of the respondents in relation to participation (n = 158)

Characteristics	Participation			p value
	Partial/none (n = 17)	Complete (n = 141)	Total (n = 158)	
	Number (%)	Number (%)	Number (%)	
Provided blood for test during MDA				
Yes	9 (52.9)	141 (100)	150 (94.9)	< *0.001*
No	8 (47.1)	0	8 (5.1)	
If yes, reasons (n = 150)				
I want to check malaria	4 (44.4)	53 (37.6)	57 (38)	0.8
I am scared of malaria	1 (11.1)	27 (19.1)	28 (18.7)	
I am scared of illness	0	10 (7.1)	10 (6.7)	
I want to be free from malaria	2 (22.2)	19 (13.5)	21 (14)	
I want to have a good health	1 (11.1)	25 (17.7)	26 (17.3)	
Other	1 (11.1)	7 (5)	8 (5.3)	
Took medicine for mass drug administration				
Yes	9 (52.9)	141 (100)	150 (94.9)	< *0.001*
No	8 (47.1)	0	8 (5.1)	
If yes, reasons (n = 150)				
I want to be free from malaria	5 (55.6)	61 (43.3)	66 (44)	0.39
I want to have a good health	1 (11.1)	54 (38.3)	55 (36.7)	
I am scared of malaria	1 (11.1)	15 (10.6)	16 (10.7)	
I am scared of illness	1 (11.1)	5 (3.5)	6 (4)	
Other	1 (11.1)	6 (4.3)	7 (4.7)	
If yes, location of the MDA (n = 150)				
Village hall	7 (77.8)	101 (71.6)	108 (72)	0.56
Village center	2 (22.2)	18 (12.8)	20 (13.3)	
Other	0	20 (14.2)	20 (13.3)	
No response	0	2 (1.4)	2 (1.3)	
Medicine distribution center was convenient				
Yes	9 (100)	138 (97.9)	147 (98)	0.83
No	0	3 (2.1)	3 (2)	
Distance between the medicine distribution center and your house (m)				
≤ 100	4 (40)	99 (70.2)	103 (68.2)	0.055
≥ 101	6 (60)	42 (29.8)	48 (31.8)	
Number of people in your household				
≤ 6	10 (58.8)	80 (56.7)	90 (57)	0.54
≥ 7	7 (41.2)	61 (43.3)	68 (43)	
Everyone in my house participated in TME				
Yes	4 (23.5)	81 (57.4)	85 (53.8)	*0.008*
No	13 (76.5)	60 (42.6)	73 (46.2)	
I had complaints after taking medicine				
Yes	3 (33.3)	27 (19.1)	30 (20)	0.25
No	6 (66.7)	114 (80.9)	120 (80)	
If yes, complaints started after				
Round 1	1 (33.3)	24 (88.9)	25 (83.3)	*0.041*
Round 2	1 (33.3)	2 (7.4)	3 (10)	
Round 3	1 (33.3)	1 (3.7)	2 (6.7)	
Household members had complaints after taking medicine (n = 153)				
Yes	3 (23.1)	36 (25.7)	39 (25.5)	*0.012*
No	9 (69.2)	103 (73.6)	112 (73.2)	
No one took the medicine	1 (7.7)	0	1 (0.7)	
Don’t know	0	1 (0.7)	1 (0.7)

**Table 4:** Perceptions on TME of the respondents in relation to participation (n = 158)

Characteristics	Participation			p value
	Partial/none (n = 17)	Complete (n = 141)	Total (n = 158)	
	Number (%)	Number (%)	Number (%)	
Received enough information about the TME				
Yes	9 (52.9)	137 (97.2)	146 (92.4)	< *0.001*
Don’t know	8 (47.1)	4 (2.8)	12 (7.6)	
Purpose of the medicine given to villagers^a^				
To kill malaria parasite in our body	8 (47.1)	132 (93.6)	140 (88.6)	< *0.001*
To protect from malaria	10 (58.8)	111 (78.7)	121 (76.6)	0.068
Gives me strength/energy	5 (29.4)	1 (0.7)	6 (3.8)	< *0.001*
Don’t know	3 (17.6)	4 (2.8)	7 (4.4)	*0.028*
MDA medicine caused many illness in your village				
Yes	0	4 (2.8)	4 (2.5)	*0.013*
No	9 (52.9)	113 (80.1)	122 (77.2)	
Don’t know	8 (47.1)	24 (17)	32 (20.3)	
Other villagers thought that medicine caused illness				
Yes	0	4 (2.8)	4 (2.5)	0.08
No	9 (52.9)	105 (74.5)	114 (72.2)	
Don’t know	8 (47.1)	32 (22.7)	40 (25.3)	
Purpose of the blood test^a^				
To test for malaria parasite	7 (41.2)	121 (85.8)	128 (81)	< *0.001*
To test for all the diseases	0	5 (3.5)	5 (3.2)	0.56
To check if we were healthy	0	1 (0.7)	1 (0.6)	0.89
Don’t know	10 (58.8)	19 (13.5)	29 (18.4)	< *0.001*
Disliked about TME				
Blood test	2 (11.8)	4 (2.8)	6 (3.8)	0.31
Unable to go to work	0	1 (0.7)	1 (0.6)	
Inadequate incentive	0	1 (0.7)	1 (0.6)	
Other	15 (88.2)	135 (95.7)	150 (94.9)	
If other, specify				
I like all	8 (53.3)	134 (99.3)	142 (94.7)	< *0.001*
I did not participate	7 (46.7)	0	7 (4.7)	
I did not like any	0	1 (0.7)	1 ((0.7)	
I think TME is important				
Yes	8 (47.1)	135 (95.7)	143 (90.5)	< *0.001*
Don’t know	9 (52.9)	6 (4.3)	15 (9.5)	
Reason for current participation in TME^a^				
Because I wanted to get rid of malaria	8 (80)	109 (77.3)	117 (77.5)	0.6
Because I wanted to be healthy	5 (50)	83 (58.9)	88 (58.3)	0.4
Other	0	2 (1.4)	2 (1.3)	0.87
I would recommend TME to others				
Yes	5 (41.7)	38 (27)	43 (28.1)	*0.004*
No	4 (33.3)	63 (44.7)	67 (43.8)	
Don’t know	2 (16.7)	40 (28.4)	42 (27.5)	
No response	1 (8.3)	0	1 (0.7)	
Ways a villager can help in the TME program				
I don’t know how to help	5 (41.7)	29 (20.6)	34 (22.2)	0.35
I will help by participating in the project	2 (16.7)	45 (31.9)	47 (30.7)	
We all have to participate	4 (33.3)	58 (41.1)	62 (40.5)	
Other	1 (8.3)	9 (90)	10 (6.5)	

^a^Multiple answers were possible, therefore percentage exceeds 100; analysis were made between “Yes” and “No”

### Factors affecting participation in TME using a logistic regression model

Variables relevant to the research question underwent univariate and multivariate logistic regression analysis. Among these, five variables were found to influence participation independently: (1) Attending TME meetings [AOR = 12.0 (95% CI 1.1–20.5) (p = 0.03)]. Those who attended meetings or events, such as audio-visual shows and poster presentations were categorized as those attending meetings or events of TME. (2) Understanding that blood tests were for the diagnosis of malaria [AOR = 5.6 (95% CI 1.0–32.3) (p = 0.05)]. Respondents had multiple options (such as through blood test, through the symptoms such as fever, chills and headache, through health worker and from the history of visiting forest) in response to how they could identify a person with malaria. TME’s health messages were focused on diagnosis of malaria using blood test, also one of the main component of TME. (3) Coming from households in which all members participated [AOR = 4.2 (95% CI 1.3–14.0) (p = 0.02)]. Respondents were asked if everyone in their household participated in MDA. Respondents were more likely to complete the MDA rounds, if all family members participated. (4) Liking all aspects of the MDA [AOR = 17.2 (95% CI 1.6–177.9) (p = 0.02)]. Respondents were asked if there were any aspects of MDA that they disliked, such as blood test, taking medicine, lack of adequate health services provided by TME, loss of work while engaged in MDA, inadequate incentive, long waiting time in queue and other dislikes. Respondents who answered “I liked all” were classified as “liking all aspects of MDA”. (5) The perception that MDA was worthwhile [AOR = 14.9 (95% CI 1.3–171.2) (p = 0.03)] (Table 5). Respondents were asked if they thought that MDA was important. Follow up questions were asked to provide the reasons; most respondents who described the importance of MDA provided reasons such as the health benefits of taking medicine, specifically to cure the disease and to avoid malaria in future.

**Table 5:** Logistic regression on association between covariates with complete participation

Covariates	Participation		Univariate analysis	p value	Multivariate analysis	p value
	Partial/none (n = 17)	Complete (n = 141)	Crude OR (95% CI)		AOR (95% CI)	
	Number (%)	Number (%)				
Sensitization by District Health Team/Village Health Workers/Study Staffs	13 (8.7)	137 (91.3)	10.53 (2.35–47.14)	*0.002*	0.98 (0.04–20.54)	0.99
Attended meetings of TME	11 (7.4)	138 (92.6)	25.09 (5.51–114.24)	< *0.001*	12.01 (1.14–125.99)	*0.03*
Village head explained TME to you	10 (7.5)	124 (92.5)	5.1 (1.71–15.19)	0.003	4.54 (0.94–21.75)	0.058
Study staffs explained TME up to 30 times	9 (6.4)	132 (93.6)	11.07 (3.65–33.61)	< *0.001*	2.97 (0.58–15.19)	0.19
We get malaria from mosquito	14 (9.2)	139 (90.8)	9.26 (1.21–70.63)	0.032	0.12 (0.002–7.60)	0.32
Fever is the sign and symptoms of malaria	8 (6.5)	115 (93.5)	4.97 (1.75–14.12)	*0.003*	2.21 (0.46–10.63)	0.32
Malaria can be diagnosed through blood test	10 (7.2)	128 (92.8)	6.89 (2.24–21.16)	*0.001*	5.68 (1.00–32.30)	*0.05*
A healthy looking person can have malaria	3 (4.8)	60 (95.2)	3.45 (0.95–12.56)	0.06	0.97 (0.18–5.15)	0.97
Malaria can be eliminated by giving medicine to all the villagers	6 (4.9)	117 (95.1)	8.93 (3.01–26.51)	< *0.001*	3.87 (0.74–20.08)	0.1
Everyone from my house participated	4 (4.7)	81 (95.3)	4.38 (1.36–14.12)	*0.013*	4.27 (1.3–14.02)	*0.017*
Had complaints after round 1	1 (4)	24 (96)	3.28 (0.41–25.94)	0.26	3.01 (0.33–26.97)	0.32
Had complaints with my HH members	3 (7.7)	36 (92.3)	1.6 (0.43–5.88)	0.48	0.89 (0.21–3.73)	0.88
Received enough information	9 (6.2)	137 (93.8)	30.44 (7.68–120.62)	< *0.001*	0.37 (0.01–11.89)	0.58
Medicine was given to kill malaria parasites	8 (5.7)	132 (94.3)	16.5 (5.13–53.02)	< *0.001*	6.77 (0.89–51.5)	0.06
Medicine did not cause many illnesses	9 (7.4)	113 (92.6)	0.27 (0.09–0.78)	0.016	1.16 (0.12–10.91)	0.89
Blood was taken to test for malaria parasite	7 (5.5)	121 (94.5)	8.64 (2.94–25.33)	< 0.001	0.76 (0.04–11.75)	0.84
I liked all about MDA	8 (5.6)	134 (94.4)	21.53 (6.36–72.82)	< 0.001	17.2 (1.66–177.99)	*0.017*
MDA is important	8 (5.6)	135 (94.4)	25.31 (7.21–88.81)	< *0.001*	14.94 (1.3–171.27)	*0.03*
I will participate if MDA happens next year	8 (5.8)	130 (94.2)	13.29 (4.27–41.32)	< 0.001	2.34 (0.27–20.05)	0.43
I would recommend MDA to others	5 (11.6)	38 (88.4)	0.88 (0.29–2.68)	0.83	0.18 (0.03–1.05)	0.057

*AOR* adjusted odds ratio for age and sex

## Discussion

The majority of respondents participated in all three rounds of MDA, which is necessary to clear parasitaemia completely [6, 9]. This study demonstrates that contact with TME staff, particularly during the community engagement meetings, was key to participating in the MDA. Villagers were also likely to be complete participants if all other household members participated. Among the community engagement activities that accompanied the MDA, village meetings were one of the most frequent means of delivering health education to the villagers.

A minority of participants never took part in MDA (n = 8) because of fears about the blood testing. Others who could not complete the participation (n = 9), gave reasons such as travelling, busy due to work and adverse events due to the medicine. Such explanations are consistent with those offered for partial or non-participation in past MDAs in the Gambia [19–21], Vietnam [14] and the Thai–Myanmar border regions [9]. The villagers’ reasons for partial or non-participation were discussed in meetings, and those who voiced concerns about MDA were sought out and provided with additional health education during house-to-house visits [16].

As has been highlighted elsewhere, the community engagement strategy played an important role in promoting MDA coverage. For example, in Vietnam, participation in TME was also more likely among villagers who could recall that someone had explained to them “what MDA is” [14]. In Vanuatu, community engagement activities provided a forum for sharing information about the study and resolving concerns raised. This ultimately contributed to the elimination of malaria [22].

Community meetings have been an integral part of MDAs in past [7]. In The Gambia, district level government officials led village meetings in which study objectives and methods were discussed and concerns and issues raised by villagers were addressed [23]. In Indonesia, villagers chose volunteers who held monthly meetings and conducted house-to-house health education [24]. In Kenya, meetings with authorities and trained volunteers were held at different community locations, such as schools and trading centres [25]. In Nicaragua [26], Liberia [27], Cambodia [28] and Sierra Leone [29] meetings were held as part of a stepwise process of community engagement for MDA.

The community engagement and other TME activities were coordinated with volunteers from each village. Through the volunteers, the villagers were able to take an active role in deciding on and executing TME activities. Such an approach has been recognized as a major element of effective community engagement [7, 17, 18, 22] and community members taking more prominent roles in the design of community engagement had a positive impact in population coverage in a recent MDA in Cambodia [10].

In addition to the community engagement, villagers’ experience of the TME study as a whole influenced their participation. Respondents who liked all the components of TME and thought that TME was a worthwhile activity participated in the MDA. Even though study staff made the distinction between community engagement and the clinical study, villagers tended to view the range of activities as part of one “project”, which is understandable given the integrated nature of community engagement within TME. Similar findings were reported from a TME study in Myanmar where villagers and staff considered community engagement an integral part of TME [30]. Consistent with the findings from Laos, perceptions such as “MDA was important” that referred to the whole study was found to be associated with participation in The Gambia [20].

The results also indicate a role for social relationships in uptake of MDA. Villagers were more likely to be complete participants if all household members participated in the study. In Laos, a high value is placed on familial cohesion and integrity [31], and in the study villages, household hierarchies, usually led by a male household head, are important [32, 33]. There was also a tendency for conformism across households in TME villages, likely to be rooted in villagers’ *Lao Theung* identity and the traditional system of mutual help between the households [32]. As previous ethnographic research has described, *Lao Theung* communities demonstrate a system of mutual support and labour exchange between households, for example work in the field, housing construction and other daily tasks. This is often termed *“aw wan sai wan”* (to take a day and to give a day) [34]. This interdependence was reflected in the communal community decision, which villagers often expressed as *“If all participate, I will participate”.*

As well as raising awareness of the study, increasing villagers’ familiarity with malaria, and addressing misconceptions, participation in village-wide meetings also generated pressure to conform and participate. Repeated home visits and interactions with TME staff and volunteers, gestures of commensality—sharing and eating food together—and participating in their rituals also strengthened social relationships. Developing ties of this kind, which went beyond the formal researcher-respondent relationship, prompted reciprocity and encouraged participation. In Myanmar, by following the social conventions (sharing traditional foods with the villagers, participating in social activities, such as funerals and festivals), study staff were able to build social relationships and garner trust. Sometimes this meant that villagers participated in MDA in spite of lack of a clear understanding of the intervention [30]. In The Gambia, developing social relationships between researchers and participants, which were akin to familial bonds, has been recognized as key to building trust and for participation in clinical trials [35].

### Strengths and limitations

This study took place alongside a clinical trial of TME, which entailed a carefully planned programme of community engagement that began 6 months before the MDA. Such intensive community engagement may not be possible for MDAs that are part of large-scale malaria control programmes. As part of large-scale implementation, it is also unlikely that blood surveys would accompany the MDA. Further research is needed to assess the factors that influence participation in large-scale mass anti-malarial administrations.

The questionnaire used for this study, has been employed in locally adapted versions from several previous surveys of factors influencing participation in MDA. The questionnaire also underwent extensive pretesting. However, using a questionnaire alone limits the depth of information on villagers’ reactions to TME, community engagement and nature of social relationships. Additional qualitative data collection will provide a more nuanced understanding of attitudes and behaviors when offered MDA in this context. Additional qualitative data collection, particularly using observations will provide insight into whether villagers’ responses were influenced by desirability bias.

In this study, the low number of partial or non-participants limits statistical comparison and increases the likelihood of type 1 error. In addition, this low sample in one of the arms within outcome variable also affects the sensitivity and specificity of the model. Future studies with large sample size with comparable arms are required for robust statistical assessment.

## Conclusion

Participation in MDA was associated with involvement in community engagement activities, knowledge that the blood test was for malaria diagnosis, family members’ participation in TME and the perception that TME was worthwhile. The comprehensive community engagement strategy, which encompassed formative research, involved villagers in implementing the study and was responsive to the needs and preferences of the community contributed to uptake of MDA in a remote population with low literacy and socio-economic status. Villagers’ overall impression of the study also influenced their participation and this illustrates that community engagement cannot be easily extricated from the overall implementation of an intervention. Social relationships were also relevant to participation in MDA, suggesting that rapid implementation that leaves little time for developing such bonds may face additional challenges. Further research is needed to investigate these factors when malaria elimination activities are scaled up.

## Additional files

**Additional file 1.** Laos TME acceptability questionnaire.

**Additional file 2.** Detailed analysis of the questionnaire.
