# Supplementary material for: Factors associated with population coverage of targeted malaria elimination (TME) in southern Savannakhet Province, Lao PDR
Source: Malar J. 2017 Oct 23;16:424. doi: 10.1186/s12936-017-2070-y (PMC5653989; doi:10.1186/s12936-017-2070-y)
Supplement: Supplementary file 1 — Additional file 1. Laos TME acceptability questionnaire. [file 12936_2017_2070_MOESM1_ESM.docx]

**Survey on Community Knowledge, experience and perceptions on TME/MDA**

| **INTRODUCTION**  My name is………………… I am a doctor/social scientist working for a research team based in Mahosot hospital in Vientiane. We are doing health research in this village as part of the project called Target Malaria Elimination in Nong District, Savannakhet Province, Laos. I am doing health research in this village. I am trying to learn perceptions and practices of villagers in relation to malaria and the targeted malaria elimination using mass administration of antimalarials. This will help to improve the malaria control programme in the future. The interview will take about 30 minutes. You can refuse to participate in the interview anytime. You can also refuse to answer some questions if you feel uncomfortable or you can stop giving answers anytime – it is your right to refuse or to participate. We will respect your refusal to participate and will not continue to ask. Your refusal will not prevent you from having normal health service or other benefits and from taking part in normal activities within the village.  The participant gives consent to be interviewed □ (Continue to ask questions)  The participant does not give consent to be interviewed □ (Please fill information in Part I and stop asking further questions) |
| --- |

PART I: INITIALS

| **Date of interview (DD/MM/YY)** | ⏐_⏐_⏐/⏐_⏐_⏐/⏐_⏐_⏐_⏐_⏐ | | |
| --- | --- | --- | --- |
| **Initials of Interviewer (First Name)** | ⏐_⏐_⏐ | | |
| **Language of Interview** | 1 Laos  2 Lao Theung  3 Other (Specify…………….) | | |
| **Individual ID** |  | | |
| **Household ID** |  | | |
| **Village ID** |  | | |
| **Name** |  | | |
| **How many rounds did you participate in MDA?** (please Tick 1 appropriate answer below) | | | |
| **Options** | **Type of participator** | **√** | **Categories/Codes** |
| 1. Not at all | Non-participator |  | 0 |
| 1. Round 1 only | Partial participators |  | 1 |
| 1. Round 2 only | Partial participators |  |  |
| 1. Round 3 only | Partial participators |  |  |
| 1. Round 1 and 3 | Partial participators |  | 2 |
| 1. Round 2 and 3 | Partial participators |  |  |
| 1. All 3 Rounds | Complete participator |  | 3 |

| **PART II: SOCIODEMOGRAPHIC CHARACTERISTICS OF RESPONDENTS (Please complete this section for all categories of respondents)** | | |
| --- | --- | --- |
|  | Respondent status in the family  1. Family Head  2. Wife of Family Head  3. Family Seniority (Specify………….)  4. Other (specify)…………… | |
|  |  | |
|  | Age (years) …………………….. | |
|  |  | |
|  | Sex | \| Male \|  \| \| --- \| --- \|  \| Female \|  \| \| --- \| --- \|  \| Other \|  \| \| --- \| --- \| |
|  |  | |
|  | Ethnicity  1. Laoloum (Specify…………..)  2. Lao Theung (Specify…………..)  3. Laosung (Specify…………..)  4. Others (Specify…………..) | |
|  |  | |
|  | What is your religion?  1. Animist  2. Buddhist  3. Christian  4. Other: (Specify………………..) | |
|  |  | |
|  | Marital status  1. Single  2. Married  3. Widow/widower  4. Divorced/Separated  5. Other (Specify…………………) | |
|  |  | |
|  | Can you read and write Lao?  1. Yes, I can read and write  2. I can read but cannot write  3. No, I cannot read nor write | |
|  |  | |
|  | Which level did you finish your normal education?  *Please fill years of education as 0, if not attended at all*  1. Years of education…………………..(years) | |
|  |  | |
|  | Occupation  1. Farmer  2. Student  3. Trader/Business  4. Civil servant (Specify……………)  5. Labor/Manual worker  6. Unemployed  7. Driver  8. Retired  9. Other: Specify………………… | |
|  |  | |
|  | How much income does your family make a month?  1. ≤500,000  2. 500,001 to 1,000,000  3. 1,000,001 to 2,000,000  4. 2,000,001 to 3,000,000  5. ≥3,000,001  6. Don’t know | |
|  |  | |
|  | What is your family daily expense?  ……………………..Kip/day | |
|  |  | |
|  | Is the amount of income enough to sustain living?  1. Yes  2. No  3. Not sure or do not know | |
|  |  | |
|  | What do you own?  *(multiple options possible)*  1.House  2.Land for farming  3.Motorbikes  4.Bicycles  5.Tractor  6.Cars  7.Cattles  8.Televisions  9.Radio  10.Mobile phones  11.Generators | |
|  |  | |
|  | What are the walls of your home made from? (Please tick it based on your observation)  *Multiple options are possible*  1. Bamboo or grass or leaves  2. Wood  3. Mud  4. Concrete  5. Brick  6. Plastic Sheeting  7. Metal or CI sheet  8. Other (Specify……………..)  9. Don’t know | |
|  |  | |
|  | What is the roof of your home made from? (Please tick it based on your observation)  *Multiple options are possible*  1. Bamboo or grass or leaves  2. Wood  3. Mud  4. Concrete  5. Brick  6. Plastic Sheeting  7. Metal or CI sheet  8. Shingles  9. Other (Specify……………..)  10. Don’t know | |
|  |  | |
|  | What is the floor of your home made from? (Please tick it based on your observation)  *Multiple options are possible*  1. Bamboo or grass or leaves  2. Wood  3. Mud or soil  4. Concrete/cement  5. Brick  6. Plastic Sheeting  7. Metal  8. Tiles  9. Other (Specify……………..)  10. Don’t know | |
|  |  | |
|  | Do you have toilet facility at home?  1.Yes  2.No | |
|  |  | |
| 17.1 | *If No,* How or where do you defecate?  *(multiple options possible)*  1. Backyard  2. In the field  3. In the forest  4. In the river or stream  5. Other (Specify…………………..) | |
|  |  | |
| 17.2 | *If Yes,* What kind of toilet facility do you have?  *(multiple options possible)*  1. Flush toilet  2. Pit latrine  3. Vault toilet  4. Other (Specify…………….)  5. Don’t know | |
|  |  | |
|  | Did you migrate from any other village?  1. Yes  2. No | |
|  |  | |
| 18.1 | *If Yes,* How long have you been living here?  1.……………… Years | |
|  |  | |
|  | How far is the forest from your house?  1………………. Km  2……………….Mins | |
|  |  | |
|  | How far is your/your family’s rice field or plantation farm from your house?  1.……………….Km  2. ………………Min  3. I don’t have land | |
|  |  | |
|  | How often do you go to forest?  1. Every day  2. Every alternate day  3. Weekly  4. Every 2 weeks  5. Every month  6. Could not specify  7. Not at all | |
|  | | |

| **PART III: KNOWLEDGE ABOUT MDA AND MALARIA** | | | | | | | | | | | | | |
| --- | --- | --- | --- | --- | --- | --- | --- | --- | --- | --- | --- | --- | --- |
|  | Have you heard/known about the current Malaria Elimination Project in your village? (TICK **ONE** RESPONSE)  1. Yes  2. No  3. Don’t know/Not sure | | | | | | | | | | | | |
|  |  | | | | | | | | | | | | |
| 22.1 | *If No/Don’t know/Not sure,* Why? Specify………………..  **(Then stop asking further questions)** | | | | | | | | | | | | |
|  |  | | | | | | | | | | | | |
|  | How did you hear about the Malaria Elimination Project? (Multiple Answers possible)  1. Sensitization by District Health Team / Village Health Workers / Study Staff  2. Flyers / banners/ posters  3. Radio or other media  4. Household member  5. Neighbor  6. Village head  7. Friend/another villager  8. Don’t know  9. No response  10. Other (Specify…………..) | | | | | | | | | | | | |
|  |  | | | | | | | | | | | | |
|  | Did you discuss the information given to you about the TME/MDA with any other person? (TICK ONE RESPONSE)  1. Yes  2. No  3. Don’t know  4. No Response | | | | | | | | | | | | |
|  |  | | | | | | | | | | | | |
| 24.1 | *If Yes,* with whom?  1. Household member  2. Relatives  3. Neighbors/other villagers/Friends  4. No one  5. Don’t know/don’t remember  6. Other (Specify……………………….) | | | | | | | | | | | | |
|  |  | | | | | | | | | | | | |
|  | Did you attend any meeting/event conducted by Malaria Elimination Project?  1. Yes  2. No/Never  3. Don’t know | | | | | | | | | | | | |
|  |  | | | | | | | | | | | | |
| 25.1 | *If No,* Why?  1. I was busy with my work/I was in the field  2. I was away from the village/I came here recently  3. I was sick  4. I was not interested in the TME/MDA  5. I did not want to participate  6. Other (Specify………………………) | | | | | | | | | | | | |
|  |  | | | | | | | | | | | | |
| 25.2 | *If Yes,* did you express an opinion/concern or ask any question in the meeting?  1. Yes  2. No  3. I don’t remember  4. Don’t know | | | | | | | | | | | | |
|  |  | | | | | | | | | | | | |
| 25.2.1 | *If Yes,* What was your concern/question? (Multiple Answers possible)  1. Taking medicine  2. Side effects of medicine  3. Giving blood  4. Duration of the project is long  5. Other (Specify………………….) | | | | | | | | | | | | |
|  |  | | | | | | | | | | | | |
| 25.2.2 | *If No,* Why? (Multiple Answers possible)  1. I felt shy  2. I did not have any question  3. I did not understand what they said  4. Don’t know  5. Other (Specify………………….) | | | | | | | | | | | | |
|  |  | | | | | | | | | | | | |
|  | Who explained the MDA/TME to you? (Multiple Answers possible)  1. Village Head  2. Village Volunteers  3. TME/MDA staffs  4. District Administrator/District Health Team  5. Neighbor/Another Villager  6. No one  7. Don’t know  8. No response  9. Other Specify (……………………..) | | | | | | | | | | | | |
|  |  | | | | | | | | | | | | |
| 26.1 | *If someone explained,* how many times did study staffs* came to you to talk about TME/MDA?  ***Study staffs includes core TME/MDA staffs from outside the village and local staffs such as village head and MDA volunteers**  1. Specify in number (…………………….)  2. Can’t remember/Don’t know | | | | | | | | | | | | |
|  |  | | | | | | | | | | | | |
|  | How many times did people not related to the study (friends, family, neighbors) explained to you about the TME/MDA?  1. Specify in number (…………………….)  2. Can’t remember/Don’t know | | | | | | | | | | | | |
|  | **Based on what you heard/learnt about TME/MDA, I would like to ask you some questions below:** | | | | | | | | | | | | |
| 27.1 | What causes malaria? (Multiple Answers possible)  1. Animals  2. Mosquito  3. Spirit  4. Germs  5. Other (Specify……………..) | | | | | | | | | | | | |
|  |  | | | | | | | | | | | | |
| 27.2 | How can we get malaria? (From) (Multiple Answers possible)  1. Air  2. Water  3. Soil  4. Forest  5. Germ  6.Worm  7. Mosquito bite  8. God/Spirit  9. Uncleaned surroundings  10. Don’t know  11. Other. (Specify……………….) | | | | | | | | | | | | |
|  |  | | | | | | | | | | | | |
| 27.3 | What are the signs and symptoms of malaria? (Multiple Answers possible)  1. Fever  2. Headache  3. Muscle pain  4. Vomiting  5. Chills/shivering  6. Sweating  7. Diarrhea  8. Sore throat  9. Jaundice  10. Running nose  11. Other (Specify…………)  1.2. Don’t know | | | | | | | | | | | | |
|  |  | | | | | | | | | | | | |
| 27.4 | How do we know if a person has malaria? (Multiple Answers possible)  1. Through blood test  2. That person will have fever, chills and headache  3. Went to see health worker  4. Went to the forest before  5. Others (specify)…………….. | | | | | | | | | | | | |
|  |  | | | | | | | | | | | | |
| 27.5 | Do you think a person in your village can have malaria parasite in his/her body without being sick?  1. Yes  2. No  3. Don’t know | | | | | | | | | | | | |
|  |  | | | | | | | | | | | | |
| 27.6 | How can we eliminate malaria from your village? (Multiple Answers possible)  1. By giving medicines to all the people in the village  2. By using mosquito nets  3. By taking regular medicine  4. By cleaning the surrounding  5. By using traditional Lao medicine  6. Witchcraft  7. Don’t know  8. Other (Specify…………….) | | | | | | | | | | | | |
| **PART IV: EXPERIENCES ON MDA/TME (For all kinds of participators, Round 1, 2 and 3)**  **For partial participators and non-participators: Go to Question no. 31** | | | | | | | | | | | | | |
|  | Did you give blood for check-up during MDA?  1. Yes  2. No  3. Don’t know | | | | | | | | | | | | |
|  |  | | | | | | | | | | | | |
| 28.1 | *If Yes,* Why? | | | | | | | | | | | | |
|  |  | | | | | | | | | | | | |
|  | Did you take medicine for mass drug administration?  1. Yes  2. No  3. Don’t know | | | | | | | | | | | | |
|  |  | | | | | | | | | | | | |
| 29.1 | *If Yes,* Why? | | | | | | | | | | | | |
|  |  | | | | | | | | | | | | |
| 29.2 | *If Yes,* Where did you take the medicine?  1. Village Hall  2. Health Center  3. School  4. Village center  5. Delivered to household (Why? ________________________________)  6. Other  7. No Response | | | | | | | | | | | | |
|  |  | | | | | | | | | | | | |
| 29.3 | Was the medicine distribution center convenient for you?  1. Yes (Why? ___________________________________________)  2. No (Why? ____________________________________________)  3. Don’t know | | | | | | | | | | | | |
|  |  | | | | | | | | | | | | |
| 29.4 | Please fill the distance between medicine distribution center from your home? (Interviewer may fill up the approximate distance between the household to the distribution center)  1. (Specify……………….Meters)  2. (Specify……………….Kilometers) | | | | | | | | | | | | |
|  |  | | | | | | | | | | | | |
| 29.5 | Please fill the time it takes to reach the medicine distribution center? (Interviewer may fill up the approximate time it takes to reach the distribution center)  1. (Specify…………………Minutes)  2. (Specify…………………Hours) | | | | | | | | | | | | |
|  |  | | | | | | | | | | | | |
|  | Did you make your own decision to take the medicine?  1. Yes  2. No  3. Don’t know | | | | | | | | | | | | |
|  |  | | | | | | | | | | | | |
| 30.1 | *If No*, Who made the decision for you to take the medicine?  1. Household head  2. Household member  3. Relatives  4. Neighbors/villagers  5. Village head  6. Village senior  7. TME team member  8. Other (Specify…………………….) | | | | | | | | | | | | |
|  | | | | | | | | | | | | | |
|  | Why did you not take the full dose/any dose of MDA medicine? (TICK **ALL** RESPONSES) **(For Partial participators and non-participators)** | | | | | | | | | | | | |
|  | OPTION (Interviewer reads out the options below) | | | | | | | YES | | | | NO | DON’T KNOW |
|  | 1. You did not want to take the medicine | | | | | | |  | | | |  |  |
|  | 2. You were travelling / you were not in the village | | | | | | |  | | | |  |  |
|  | 3. You do not trust the MDA / You are not happy with the MDA / You feel that you are being used by the MDA | | | | | | |  | | | |  |  |
|  | 4. You have never taken any medicines | | | | | | |  | | | |  |  |
|  | 5. You only take medicines when you are sick | | | | | | |  | | | |  |  |
|  | 6. Malaria is not a problem for you | | | | | | |  | | | |  |  |
|  | 7. You did not know what the medicine was for | | | | | | |  | | | |  |  |
|  | 8. Other people became sick after taking the medicine | | | | | | |  | | | |  |  |
|  | **9. You were told not to take the medicine** | | | | | | |  | | | |  |  |
|  | 10. You did not have time / You were working/busy | | | | | | |  | | | |  |  |
|  | 11. You only take traditional medicine | | | | | | |  | | | |  |  |
|  | 12. You are old | | | | | | |  | | | |  |  |
|  | 13. You were taking another medicine at the same time | | | | | | |  | | | |  |  |
|  | 14. You were pregnant | | | | | | |  | | | |  |  |
|  | You were allergic or had adverse events after taking the MDA drugs | | | | | | |  | | | |  |  |
|  | 15. Don’t know | | | | | | |  | | | |  |  |
|  | 16. No Response | | | | | | |  | | | |  |  |
|  | 17. Other (Specify……………..) | | | | | | | | | | | | |
|  |  | | | | | | | | | | | | |
| 31.1 | *If You were told not to take the medicine:* Who told you?  (Specify…………… | | | | | | | | | | | | |
|  |  | | | | | | | | | | | | |
| 31.2 | *If You were told not to take the medicine:* Why did they tell you?  (Specify……………………….. | | | | | | | | | | | | |
|  |  | | | | | | | | | | | | |
|  | How many people are there in your household?  1. Specify (………......)  2. Don’t know  3. No response | | | | | | | | | | | | |
|  |  | | | | | | | | | | | | |
|  | Did everyone in your household take medicine/participate in the MDA/TME?  1. Yes  2. No  3. Don’t know  4. No Response | | | | | | | | | | | | |
|  |  | | | | | | | | | | | | |
| 33.1 | *If No,* how many individuals in your household did not take medicine/participate in the MDA/TME?  1. (Specify…………………..)  2. Don’t know  3. No Response | | | | | | | | | | | | |
|  | **(For all kinds of participators)** | | | | | | | | | | | | |
|  | Did you have any problems/complaints after taking the medicine?  1. Yes  2. No  3. Don’t Know  4. No Response | | | | | | | | | | | | |
|  |  | | | | | | | | | | | | |
| 34.1 | *If Yes,* When did the problems/complaints start? (Please tick the appropriate boxes) | | | | | | | | | | | | |
|  | Round 1 | | | | Round 2 | | | | Round 3 | | | | |
|  | Day 1 | Day 2 | Day 3 | | Day 1 | Day 2 | Day 3 | | Day 1 | | Day 2 | | Day 3 |
|  |  |  |  | |  |  |  | |  | |  | |  |
|  | 1. Specify (After round……….., after day…………. Until day………………….)  2. Don’t know  3. No Response | | | | | | | | | | | | |
|  |  | | | | | | | | | | | | |
|  | What complaints did you have after taking the medicine? (TICK **ALL** RESPONSES) | | | | | | | | | | | | |
|  | OPTION | | | YES | *If Yes,* how long did the complaints last? | | | | | | | NO | DON’T KNOW |
|  | General | | |  |  | | | | | | |  |  |
|  | 1. Weakness  2. Headache  3. Fever  4. Malaise | | |  | 1. Hours (Specify………...)  2. Days (Specify…………)  3. Don’t know  4. No Response | | | | | | |  |  |
|  | Respiratory | | |  |  | | | | | | |  |  |
|  | 1. Cough  2. Difficulty breathing  3. Chest pain  4. Other (Specify……..) | | |  | 1. Hours (Specify………...)  2. Days (Specify…………)  3. Don’t know  4. No Response | | | | | | |  |  |
|  | Neurological | | |  |  | | | | | | |  |  |
|  | 1. Dizziness  2. Vertigo  3. Paresthesia  4. Paralysis | | |  | 1. Hours (Specify………...)  2. Days (Specify…………)  3. Don’t know  4. No Response | | | | | | |  |  |
|  | Gastro Intestinal | | |  |  | | | | | | |  |  |
|  | 1. Loss of appetite  2. Dyspepsia  3. Vomiting  4. Diarrhea  5. Pain abdomen | | |  | 1. Hours (Specify………...)  2. Days (Specify…………)  3. Don’t know  4. No Response | | | | | | |  |  |
|  | Dermatological | | |  |  | | | | | | |  |  |
|  | 1. Itching  2. Rash | | |  | 1. Hours (Specify………...)  2. Days (Specify…………)  3. Don’t know  4. No Response | | | | | | |  |  |
|  | 7. Don’t Know | | |  |  | | | | | | |  |  |
|  | 8. No Response | | |  |  | | | | | | |  |  |
|  | 9. Other (Specify……………………………………………………) | | | | | | | | | | | | |
|  | | | | | | | | | | | | | |
|  | Did anyone in your household have any complaints after taking the medicine? (TICK **ONE** RESPONSE)  1. Yes  2. No  3. N/A (No one else took the medicine)  4. Don’t know  5. No Response | | | | | | | | | | | | |
|  |  | | | | | | | | | | | | |
| 36.1 | *If Yes,* how many individuals in your household had any complaints after taking the medicine? (TICK **ONE** RESPONSE)  1. Specify (…………………………)  2. Don’t know  3. No Response | | | | | | | | | | | | |
|  |  | | | | | | | | | | | | |
| 36.2 | *If Yes,* Please fill the details | | | | | | | | | | | | |
| 1 | First name: Last name:  Age:  Sex:  Relationship with respondent:  Complaints:   \| 1. Loss of appetite \|  \| \| --- \| --- \| \| 2. Headache \|  \| \| 3. Epigastric pain \|  \| \| 4. Tiredness/weakness \|  \| \| 5. Rash \|  \| \| 6. Fever \|  \| \| 7. Cough \|  \| \| 8. Nausea/vomiting \|  \| \| 9. Other (Specify) \| \| | | | | | | | | | | | | |
| 2 | First name: Last name:  Age:  Sex:  Relationship with respondent:  Complaints:   \| 1. Loss of appetite \|  \| \| --- \| --- \| \| 2. Headache \|  \| \| 3. Epigastric pain \|  \| \| 4. Tiredness/weakness \|  \| \| 5. Rash \|  \| \| 6. Fever \|  \| \| 7. Cough \|  \| \| 8. Nausea/vomiting \|  \| \| 9. Other (Specify) \| \| | | | | | | | | | | | | |
| 3 | First name: Last name:  Age:  Sex:  Relationship with respondent:  Complaints:   \| 1. Loss of appetite \|  \| \| --- \| --- \| \| 2. Headache \|  \| \| 3. Epigastric pain \|  \| \| 4. Tiredness/weakness \|  \| \| 5. Rash \|  \| \| 6. Fever \|  \| \| 7. Cough \|  \| \| 8. Nausea/vomiting \|  \| \| 9. Other (Specify) \| \| | | | | | | | | | | | | |
| 4 | First name: Last name:  Age:  Sex:  Relationship with respondent:  Complaints:   \| 1. Loss of appetite \|  \| \| --- \| --- \| \| 2. Headache \|  \| \| 3. Epigastric pain \|  \| \| 4. Tiredness/weakness \|  \| \| 5. Rash \|  \| \| 6. Fever \|  \| \| 7. Cough \|  \| \| 8. Nausea/vomiting \|  \| \| 9. Other (Specify) \| \| | | | | | | | | | | | | |
| 5 | First name: Last name:  Age:  Sex:  Relationship with respondent:  Complaints:   \| 1. Loss of appetite \|  \| \| --- \| --- \| \| 2. Headache \|  \| \| 3. Epigastric pain \|  \| \| 4. Tiredness/weakness \|  \| \| 5. Rash \|  \| \| 6. Fever \|  \| \| 7. Cough \|  \| \| 8. Nausea/vomiting \|  \| \| 9. Other (Specify) \| \| | | | | | | | | | | | | |
| 6 | First name: Last name:  Age:  Sex:  Relationship with respondent:  Complaints:   \| 1. Loss of appetite \|  \| \| --- \| --- \| \| 2. Headache \|  \| \| 3. Epigastric pain \|  \| \| 4. Tiredness/weakness \|  \| \| 5. Rash \|  \| \| 6. Fever \|  \| \| 7. Cough \|  \| \| 8. Nausea/vomiting \|  \| \| 9. Other (Specify) \| \| | | | | | | | | | | | | |
| 7 | First name: Last name:  Age:  Sex:  Relationship with respondent:  Complaints:   \| 1. Loss of appetite \|  \| \| --- \| --- \| \| 2. Headache \|  \| \| 3. Epigastric pain \|  \| \| 4. Tiredness/weakness \|  \| \| 5. Rash \|  \| \| 6. Fever \|  \| \| 7. Cough \|  \| \| 8. Nausea/vomiting \|  \| \| 9. Other (Specify) \| \| | | | | | | | | | | | | |
| **PART V: PERCEPTIONS ON MDA (For both participators and non-participators)** | | | | | | | | | | | | | |
|  | Do you think you received enough information about the MDA/TME?  1. Yes  2. No (Why? _________________________________________________  3. Don’t know  4. No Response | | | | | | | | | | | | |
|  |  | | | | | | | | | | | | |
|  | What do you think was the medicine given to villagers for? (Multiple Answers possible)  1. To kill malaria parasite in our body  2. To protect from Malaria  3. Mosquito will not be able to bite me  4. I don’t need to sleep under bed net after taking the medicine  5. Gives me strength/energy  6. Don’t know  7. No Response  8. Other (Specify……………………….) | | | | | | | | | | | | |
|  |  | | | | | | | | | | | | |
|  | Do you think that the medicine provided to villagers during MDA caused many illnesses or problems in your village?  1. Yes  2. No  3. Don’t know | | | | | | | | | | | | |
|  |  | | | | | | | | | | | | |
|  | Did you hear if any other villagers thought that the medicine provided to villagers during MDA caused many illnesses or problems in your village?  1. Yes  2. No  3. Don’t know | | | | | | | | | | | | |
|  |  | | | | | | | | | | | | |
|  | What do you think the blood was taken for? (Multiple Answers possible)  1. To test for malaria parasite  2. To test for all the diseases  3. To check if we were healthy  4. To clean our blood from diseases  5. The blood was taken for sell  6. Don’t know  7. Other (Specify…………….) | | | | | | | | | | | | |
|  |  | | | | | | | | | | | | |
|  | What did villagers think about the blood test during MDA? (Multiple Answers possible)  1. The blood test was for malaria parasite  2. The blood test was for all the diseases  3. The blood test was to check if we were healthy  4. The blood was taken to sell  5. To clean our blood from diseases  6. Don’t know  7. Other (Specify…………….) | | | | | | | | | | | | |
|  |  | | | | | | | | | | | | |
|  | Do you think the number of people who fall sick with malaria will decrease this year? (TICK **ONE** RESPONSE)  1. Yes  2. No  3. May be  4. Don’t know | | | | | | | | | | | | |
|  |  | | | | | | | | | | | | |
| 43.1 | *If Yes,* Why?  Specify (……………….. | | | | | | | | | | | | |
|  |  | | | | | | | | | | | | |
| 43.2 | *If No,* Why?  Specify (………………….  *If May be,* Why?  Specify (……………………….. | | | | | | | | | | | | |
|  |  | | | | | | | | | | | | |
|  | If a group of people take the medicine and another group does not take the medicine, what do you think will happen? | | | | | | | | | YES | | NO | DON’T KNOW |
|  | 1. Only the group that takes medicine has less malaria  2. Both groups have less malaria  3. No difference in malaria  4. Don’t Know  5. No Response | | | | | | | | |  | |  |  |
|  |  | | | | | | | | | | | | |
|  | What did you not like about MDA? (multiple options possible)  1. Blood test  2. Taking medicine  3. Lack of adequate health services to us  4. Unable to go to work during TME visit in the village  5. Inadequate incentive  6. Waiting in queue for medicine and test  7. Other (Specify _____________________________________________________ | | | | | | | | | | | | |
|  |  | | | | | | | | | | | | |
|  | Do you think the MDA/TME is important?  1. Yes (Why? __________________________________________________  2. No (Why? __________________________________________________  3. May be (If __________________________________________________  4. Don’t Know  5. No Response | | | | | | | | | | | | |
|  |  | | | | | | | | | | | | |
|  | If MDA happens again next year, would you take the medicine or participate?  1. Yes  2. No  3. Yes, only if……………………………………………………………….  4. Don’t know  5. No response | | | | | | | | | | | | |
|  |  | | | | | | | | | | | | |
|  | Can you tell us why did you participate in TME?  1. Because I wanted to get rid of malaria  2. Because I wanted to be healthy  3. Because TME provides us free health care  4. Because TME provides incentives (money) to us  5. Because TME provided milk and cake to us  6. Other (Specify………………………………………………..) | | | | | | | | | | | | |
|  |  | | | | | | | | | | | | |
|  | Which among these, if not provided in future, would you not participate in TME? (Read out the options)  1. Free health care  2. Money  3. Milk and cake  4. Mosquito nets  5. Cooking utensils  6. T-shirts  7. All of the above  8. I will participate even if TME doesn’t provide anything  9. Other (Specify……….) | | | | | | | | | | | | |
|  |  | | | | | | | | | | | | |
|  | In future, if TME/MDA doesn’t provide you the free health care, money and other materials such as cooking utensils, would you still participate? **(Provided nothing)**  1. Yes  2. No  3. Don’t know | | | | | | | | | | | | |
|  |  | | | | | | | | | | | | |
|  | In future, if TME/MDA doesn’t provide you the money but just the free health care, would you still participate? **(Only free health care)**  1. Yes  2. No  3. Don’t know | | | | | | | | | | | | |
|  |  | | | | | | | | | | | | |
|  | In future, if TME/MDA doesn’t provide you the free health care but just the money, would you still participate? **(only money)**  1. Yes  2. No  3. Don’t know | | | | | | | | | | | | |
|  |  | | | | | | | | | | | | |
|  | In future, if TME/MDA doesn’t provide you money and free health care but just some materials such as cooking utensils, would you still participate? **(only cooking utensils)**  1. Yes  2. No  3. Don’t know | | | | | | | | | | | | |
|  |  | | | | | | | | | | | | |
|  | Would you recommend the MDA/TME program to someone else?  1. Yes (Why? __________________________________________________  2. No (Why? __________________________________________________  3. May be (If __________________________________________________  4. Don’t Know  5. No Response | | | | | | | | | | | | |
|  |  | | | | | | | | | | | | |
|  | How do you think the village can help in the TME program?  Specify (_______________________________________________________ | | | | | | | | | | | | |
| **The End** | | | | | | | | | | | | | |
