# Supplementary material for: Factors associated with population coverage of targeted malaria elimination (TME) in southern Savannakhet Province, Lao PDR
Source: Malar J. 2017 Oct 23;16:424. doi: 10.1186/s12936-017-2070-y (PMC5653989; doi:10.1186/s12936-017-2070-y)
Supplement: Supplementary file 2 — Additional file 2. Detailed analysis of the questionnaire. [file 12936_2017_2070_MOESM2_ESM.docx]

| **Table S1: Socio-demographic and economic characteristics of the respondents in relation to participation (n=158)** | | | | |
| --- | --- | --- | --- | --- |
|  | **Participation** |  |  |  |
| **Characteristics** | **Partial/none (n=17)** | **Complete (n=141)** | **Total (n=158)** | **p-value** |
|  | **Number (%)** | **Number (%)** | **Number (%)** |  |
| **Respondent status** |  |  |  |  |
| Family Head | 12 (70.6) | 88 (62.4) | 100 (63.3) | 0.35 |
| Other | 5 (29.4) | 53 (37.6) | 58 (36.7) |  |
| **Age group** |  |  |  |  |
| ≤29 years | 6 (35.3) | 47 (33.3) | 53 (33.5) | 0.72 |
| 30-40 years | 7 (41.2) | 48 (34) | 55 (34.8) |  |
| ≥41 years | 4 (23.5) | 46 (32.6) | 50 (31.6) |  |
| **Sex** |  |  |  |  |
| Female | 5 (29.4) | 29 (20.6) | 34 (21.5) | 0.28 |
| Male | 12 (70.6) | 112 (79.4) | 124 (78.5) |  |
| **Ethnicity** |  |  |  |  |
| *Lao Lum* | 1 (5.9) | 1 (0.7) | 2 (1.3) | 0.16 |
| *Lao Theung* | 16 (94.1) | 137 (97.2) | 153 (96.8) |  |
| Other | 0 | 3 (2.1) | 3 (1.9) |  |
| **Religion** |  |  |  |  |
| Animist | 16 (94.1) | 138 (97.9) | 154 (97.5) | 0.36 |
| Buddhist | 1 (5.9) | 3 (2.1) | 4 (2.5) |  |
| **Marital Status** |  |  |  |  |
| In relationship | 17 (100) | 129 (91.5) | 146 (92.4) | 0.24 |
| Not in relationship | 0 | 12 (8.5) | 12 (7.6) |  |
| **Literacy** |  |  |  |  |
| Illiterate | 14 (82.4) | 101 (71.6) | 115 (72.8) | 0.26 |
| Literate | 3 (17.6) | 40 (28.4) | 43 (27.2) |  |
| **Education in years** |  |  |  |  |
| ≤5 years | 16 (94.1) | 127 (90.1) | 143 (90.5) | 0.5 |
| ≥5.1 years | 1 (5.9) | 14 (9.9) | 15 (9.5) |  |
| **Occupation** |  |  |  |  |
| Farmer | 16 (94.1) | 131 (92.9) | 147 (93) | 0.66 |
| Other | 1 (5.9) | 10 (7.1) | 11 (7) |  |
| **Monthly income** |  |  |  |  |
| ≤500,000 kip | 16 (94.1) | 124 (87.9) | 140 (88.6) | 0.72 |
| ≥500,001 kip | 1 (5.9) | 15 (10.6) | 16 (10.1) |  |
| Don't know | 0 | 2 (1.4) | 2 (1.3) |  |
| **Daily expense** |  |  |  |  |
| ≤3000 kip | 12 (70.6) | 76 (53.9) | 88 (55.7) | 0.14 |
| ≥3001 kip | 5 (29.4) | 65 (46.1) | 70 (44.3) |  |
| **Properties owned*** |  |  |  |  |
| House | 16 (94.1) | 141 (100) | 157 (99.4) | 0.1 |
| Land | 17 (100) | 135 (95.7) | 152 (96.2) | 0.49 |
| Motorbike | 10 (58.8) | 107 (75.9) | 117 (74.1) | 0.11 |
| Tractor | 6 (35.3) | 48 (34) | 54 (34.2) | 0.55 |
| Cars | 1 (5.9) | 4 (2.8) | 5 (3.2) | 0.43 |
| Cattles | 9 (52.9) | 69 (48.9) | 81 (51.3) | 0.54 |
| TV | 1 (5.9) | 31 (22) | 32 (20.3) | 0.1 |
| Radio | 0 | 8 (5.7) | 8 (5.1) | 0.39 |
| Mobile | 5 (29.4) | 56 (39.7) | 61 (38.6) | 0.29 |
| **Material of the wall*** |  |  |  |  |
| Bamboo | 6 (35.3) | 29 (20.6) | 35 (22.2) | 0.14 |
| Wood | 13 (76.5) | 113 (80.1) | 126 (79.7) | 0.46 |
| Concrete | 0 | 2 (1.4) | 2 (1.3) | 0.79 |
| Plastic | 0 | 4 (2.8) | 4 (2.5) | 0.63 |
| Metal | 0 | 3 (2.1) | 3 (1.9) | 0.7 |
| **Material of the roof*** |  |  |  |  |
| Bamboo | 1 (5.9) | 8 (5.7) | 9 (5.7) | 0.65 |
| Wood | 1 (5.9) | 1 (0.7) | 2 (1.3) | 0.2 |
| Plastic | 1 (5.9) | 3 (2.1) | 4 (2.5) | 0.36 |
| Metal | 13 (76.5) | 105 (74.5) | 118 (74.7) | 0.56 |
| Shingles | 3 (17.6) | 30 (21.3) | 33 (20.9) | 0.5 |
| **Material of the floor*** |  |  |  |  |
| Bamboo | 2 (11.8) | 11 (7.8) | 13 (8.2) | 0.42 |
| Wood | 16 (94.1) | 130 (92.2) | 146 (92.4) | 0.62 |
| Mud | 0 | 3 (2.1) | 3 (1.9) | 0.7 |
| Concrete | 0 | 5 (3.5) | 5 (3.2) | 0.56 |
| **Presence of Toilet Facility at home** | |  |  |  |
| Yes | 3 (17.6) | 18 (12.8) | 21 (13.3) | 0.4 |
| No | 14 (82.4) | 123 (87.2) | 137 (86.7) |  |
| ***If No,* you defecate at (n=137)** | |  |  |  |
| Field | 0 | 2 (1.6) | 2 (1.5) | 0.8 |
| Forest | 14 (100) | 121 (98.4) | 135 (98.5) |  |
| **Migrated from another village** | |  |  |  |
| Yes | 6 (35.3) | 45 (31.9) | 51 (32.3) | 0.48 |
| No | 11 (64.7) | 96 (68.1) | 107 (67.7) |  |
| ***If Yes,* years of living (n=51)** | |  |  |  |
| ≤15 years | 4 (66.7) | 25 (55.6) | 29 (56.9) | 0.47 |
| ≥15 years | 2 (33.3) | 20 (44.4) | 22 (43.1) |  |
| **Distance between forest and house in km** | | |  |  |
| ≤1 km | 10 (62.5) | 82 (59.9) | 92 (60.1) | 0.53 |
| ≥1.1 km | 6 (37.5) | 55 (40.1) | 61 (39.9) |  |
| **Distance between rice field and house in km** | | |  |  |
| ≤2 km | 12 (75) | 89 (68.5) | 101 (69.2) | 0.414 |
| ≥2.1 km | 4 (25) | 41 (31.5) | 45 (30.8) |  |
| **Frequency of visit to forest** | |  |  |  |
| Everyday | 9 (52.9) | 87 (61.7) | 96 (60.8) | 0.77 |
| ≥ Every alternate day | 7 (41.2) | 48 (34) | 55 (34.8) |  |
| NA | 1 (5.9) | 6 (4.3) | 7 (4.4) |  |
| *Multiple answers were possible; percentage exceeds 100; analysis were made between "Yes" and "No" | | | | |

| **Table S2: Knowledge about TME and Malaria of the respondents in relation to participation (n=158)** | | | | |
| --- | --- | --- | --- | --- |
|  | **Participation** |  |  |  |
| **Characteristics** | **Partial/none (n=17)** | **Complete (n=141)** | **Total (n=158)** | **p-value** |
|  | **Number (%)** | **Number (%)** | **Number (%)** |  |
| **Heard about the current malaria elimination project** | | |  |  |
| Yes | 17 (100) | 141 (100) | 158 (100) | NA |
| **Heard through/from*** |  |  |  |  |
| District health team/village health workers/study staffs | 13 (76.5) | 137 (97.2) | 150 (94.9) | **0.005** |
| Neighbor | 0 | 1 (0.6) | 1 (0.6) | 0.89 |
| Village Head | 10 (58.8) | 109 (77.3) | 119 (75.3) | 0.089 |
| Don’t know | 1 (5.9) | 4 (2.8) | 5 (3.2) | 0.43 |
| **Discussed the information about TME with another person** | | | |  |
| Yes | 2 (11.8) | 40 (28.4) | 42 (26.6) | 0.25 |
| No | 14 (82.4) | 98 (69.5) | 112 (70.9) |  |
| Don't know | 1 (5.9) | 3 (2.1) | 4 (2.5) |  |
| **Attended meetings/events conducted by TME** | | |  |  |
| Yes | 11 (64.7) | 138 (97.9) | 149 (94.3) | **<0.001** |
| No | 6 (35.3) | 3 (2.1) | 9 (5.7) |  |
| **TME was explained to you by*** | |  |  |  |
| Village Head | 10 (58.8) | 124 (87.9) | 134 (84.8) | **0.005** |
| Volunteers | 9 (52.9) | 117 (83) | 126 (79.7) | **0.008** |
| TME staffs | 9 (52.9) | 132 (93.6) | 141 (89.2) | **<0.001** |
| **Frequency of explanation about TME by study staffs** | | |  |  |
| Up to 30 times | 8 (47.1) | 128 (90.8) | 136 (86.1) | **<0.001** |
| Can't remember/Don't know | 9 (52.9) | 13 (9.2) | 22 (13.9) |  |
| **Frequency of explanation about TME by non-study staffs** | | | |  |
| Up to 20 times | 10 (58.8) | 137 (97.2) | 147 (93) | **<0.001** |
| Can't remember/Don't know | 7 (41.2) | 4 (2.8) | 11 (7) |  |
| **Causes of malaria** |  |  |  |  |
| Mosquito | 15 (88.2) | 139 (98.6) | 154 (97.5) | 0.058 |
| Other/I don't know | 2 (11.8) | 2 (1.4) | 4 (2.5) |  |
| **We get malaria from*** |  |  |  |  |
| Forest | 2 (11.8) | 2 (1.4) | 4 (2.5) | 0.058 |
| Mosquito | 14 (82.4) | 139 (98.6) | 153 (96.8) | **0.009** |
| **Signs and symptoms of malaria*** | |  |  |  |
| Fever | 8 (47.1) | 115 (81.6) | 123 (77.8) | **0.003** |
| Headache | 7 (41.2) | 105 (74.5) | 112 (70.9) | **0.007** |
| Muscle pain | 1 (5.9) | 14 (9.9) | 15 (9.5) | 0.5 |
| Vomiting | 1 (5.9) | 7 (5) | 8 (5.1) | 0.6 |
| Chills/Shivering | 8 (47.1) | 116 (82.3) | 124 (78.5) | **0.003** |
| Diarrhea | 1 (5.9) | 7 (5) | 8 (5.1) | 0.6 |
| Don't know | 6 (35.3) | 15 (10.6) | 21 (13.3) | **0.013** |
| **Diagnosis of malaria*** |  |  |  |  |
| Through blood test | 10 (58.8) | 128 (90.8) | 138 (87.3) | **0.002** |
| That person will have fever, chills and headache | 0 | 7 (5) | 7 (4.4) | 0.44 |
| Went to health worker | 14 (82.4) | 117 (83) | 131 (82.9) | 0.58 |
| Went to forest before | 2 (11.8) | 0 | 2 (1.3) | **0.011** |
| **An asymptomatic villager can have malaria parasite** | | |  |  |
| Yes | 3 (17.6) | 60 (42.6) | 63 (39.9) | **0.04** |
| No | 1 (5.9) | 19 (13.5) | 20 (12.7) |  |
| Don't know | 13 (76.5) | 62 (44) | 75 (47.5) |  |
| **Ways to eliminate malaria from the village*** | | |  |  |
| By Giving medicine to all the villagers | 6 (35.3) | 117 (83) | 123 (77.8) | **<0.001** |
| By using mosquito nets | 1 (5.9) | 6 (4.3) | 7 (4.4) | 0.55 |
| By Cleaning the surrounding | 0 | 2 (1.4) | 2 (1.3) | 0.79 |
| Don’t know | 9 (52.9) | 18 (12.8) | 27 (17.1) | **<0.001** |
| *Multiple answers were possible; percentage exceeds 100; analysis were made between "Yes" and "No" | | | | |

| **Table S3: Experiences of TME of the respondents in relation to participation (n=158)** | | | | |
| --- | --- | --- | --- | --- |
|  | **Participation** |  |  |  |
| **Characteristics** | **Partial/none (n=17)** | **Complete (n=141)** | **Total (n=158)** | **p-value** |
|  | **Number (%)** | **Number (%)** | **Number (%)** |  |
| **Provided blood for test during MDA** | |  |  |  |
| Yes | 9 (52.9) | 141 (100) | 150 (94.9) | **<0.001** |
| No | 8 (47.1) | 0 | 8 (5.1) |  |
| ***If Yes,* reasons (n=150)** |  |  |  |  |
| I want to check malaria | 4 (44.4) | 53 (37.6) | 57 (38) | 0.8 |
| I am scared of malaria | 1 (11.1) | 27 (19.1) | 28 (18.7) |  |
| I am scared of illness | 0 | 10 (7.1) | 10 (6.7) |  |
| I want to be free from malaria | 2 (22.2) | 19 (13.5) | 21 (14) |  |
| I want to have a good health | 1 (11.1) | 25 (17.7) | 26 (17.3) |  |
| Other | 1 (11.1) | 7 (5) | 8 (5.3) |  |
| **Took medicine for mass drug administration** | |  |  |  |
| Yes | 9 (52.9) | 141 (100) | 150 (94.9) | **<0.001** |
| No | 8 (47.1) | 0 | 8 (5.1) |  |
| ***If Yes,* reasons (n=150)** |  |  |  |  |
| I want to be free from malaria | 5 (55.6) | 61 (43.3) | 66 (44) | 0.39 |
| I want to have a good health | 1 (11.1) | 54 (38.3) | 55 (36.7) |  |
| I am scared of malaria | 1 (11.1) | 15 (10.6) | 16 (10.7) |  |
| I am scared of illness | 1 (11.1) | 5 (3.5) | 6 (4) |  |
| Other | 1 (11.1) | 6 (4.3) | 7 (4.7) |  |
| ***If Yes,* location of the MDA (n=150)** | |  |  |  |
| Village hall | 7 (77.8) | 101 (71.6) | 108 (72) | 0.56 |
| Village center | 2 (22.2) | 18 (12.8) | 20 (13.3) |  |
| Other | 0 | 20 (14.2) | 20 (13.3) |  |
| No Response | 0 | 2 (1.4) | 2 (1.3) |  |
| **Medicine distribution center was convenient** | |  |  |  |
| Yes | 9 (100) | 138 (97.9) | 147 (98) | 0.83 |
| No | 0 | 3 (2.1) | 3 (2) |  |
| **Distance between the medicine distribution center and your house** | | | |  |
| ≤100 meter | 4 (40) | 99 (70.2) | 103 (68.2) | 0.055 |
| ≥101 meter | 6 (60) | 42 (29.8) | 48 (31.8) |  |
| **Number of people in your household** | |  |  |  |
| ≤6 | 10 (58.8) | 80 (56.7) | 90 (57) | 0.54 |
| ≥7 | 7 (41.2) | 61 (43.3) | 68 (43) |  |
| **Everyone in my house participated in TME** | |  |  |  |
| Yes | 4 (23.5) | 81 (57.4) | 85 (53.8) | **0.008** |
| No | 13 (76.5) | 60 (42.6) | 73 (46.2) |  |
| **I had complaints after taking medicine** | |  |  |  |
| Yes | 3 (33.3) | 27 (19.1) | 30 (20) | 0.25 |
| No | 6 (66.7) | 114 (80.9) | 120 (80) |  |
| ***If Yes,* complaints started after** | |  |  |  |
| Round 1 | 1 (33.3) | 24 (88.9) | 25 (83.3) | **0.041** |
| Round 2 | 1 (33.3) | 2 (7.4) | 3 (10) |  |
| Round 3 | 1 (33.3) | 1 (3.7) | 2 (6.7) |  |
| **Household members had complaints after taking medicine (n=153)** | | | |  |
| Yes | 3 (23.1) | 36 (25.7) | 39 (25.5) | **0.012** |
| No | 9 (69.2) | 103 (73.6) | 112 (73.2) |  |
| No one took the medicine | 1 (7.7) | 0 | 1 (0.7) |  |
| Don't know | 0 | 1 (0.7) | 1 (0.7) |  |

| **Table S4: Perceptions on TME of the respondents in relation to participation (n=158)** | | | | |
| --- | --- | --- | --- | --- |
|  | **Participation** |  |  |  |
| **Characteristics** | **Partial/none (n=17)** | **Complete (n=141)** | **Total (n=158)** | **p-value** |
|  | **Number (%)** | **Number (%)** | **Number (%)** | |
| **Received enough information about the TME** | | |  |  |
| Yes | 9 (52.9) | 137 (97.2) | 146 (92.4) | **<0.001** |
| Don't know | 8 (47.1) | 4 (2.8) | 12 (7.6) |  |
| **Purpose of the medicine given to villagers*** | | |  |  |
| To kill malaria parasite in our body | 8 (47.1) | 132 (93.6) | 140 (88.6) | **<0.001** |
| To protect from malaria | 10 (58.8) | 111 (78.7) | 121 (76.6) | 0.068 |
| Gives me strength/energy | 5 (29.4) | 1 (0.7) | 6 (3.8) | **<0.001** |
| Don't know | 3 (17.6) | 4 (2.8) | 7 (4.4) | **0.028** |
| **MDA medicine caused many illness in your village** | | |  |  |
| Yes | 0 | 4 (2.8) | 4 (2.5) | **0.013** |
| No | 9 (52.9) | 113 (80.1) | 122 (77.2) |  |
| Don't know | 8 (47.1) | 24 (17) | 32 (20.3) |  |
| **Other villagers thought that medicine caused illness** | | |  |  |
| Yes | 0 | 4 (2.8) | 4 (2.5) | 0.08 |
| No | 9 (52.9) | 105 (74.5) | 114 (72.2) |  |
| Don't know | 8 (47.1) | 32 (22.7) | 40 (25.3) |  |
| **Purpose of the blood test*** | |  |  |  |
| To test for malaria parasite | 7 (41.2) | 121 (85.8) | 128 (81) | **<0.001** |
| To test for all the diseases | 0 | 5 (3.5) | 5 (3.2) | 0.56 |
| To check if we were healthy | 0 | 1 (0.7) | 1 (0.6) | 0.89 |
| Don't know | 10 (58.8) | 19 (13.5) | 29 (18.4) | **<0.001** |
| **Purpose of the blood test thought by villagers*** | | |  |  |
| To test for malaria parasite | 4 (23.5) | 81 (57.4) | 85 (53.8) | **0.008** |
| To check all the diseases | 0 | 4 (2.8) | 4 (2.5) | 0.63 |
| To check if we were healthy | 0 | 3 (2.1) | 3 (1.9) | 0.7 |
| To sell | 17 (100) | 140 (99.3) | 1 (0.6) | 0.89 |
| To clean our blood from diseases | 1 (5.9) | 0 | 1 (0.6) | 0.1 |
| Don't know | 12 (70.6) | 54 (38.3) | 66 (41.8) | **0.011** |
| **Number of people with malaria will decrease this year** | | |  |  |
| Yes | 7 (41.2) | 90 (63.8) | 97 (61.4) | 0.18 |
| No | 0 | 2 (1.4) | 2 (1.3) |  |
| Maybe | 0 | 3 (2.1) | 3 (1.9) |  |
| Don’t know | 10 (58.8) | 46 (32.6) | 56 (35.4) |  |
| ***If yes,* reasons** |  |  |  |  |
| Because we took medicine | 6 (85.7) | 82 (91.1) | 88 (90.7) | 0.31 |
| We tested blood and took medicine | 0 | 5 (5.6) | 5 (5.2) |  |
| Other | 1 (14.3) | 3 (3.3) | 4 (4.1) |  |
| **If only a group of people take medicine, consequences are** | | | |  |
| Medicated group will have less malaria | 5 (29.4) | 86 (61) | 91 (57.6) | 0.07 |
| Both groups will have less malaria | 0 | 1 (0.7) | 1 (0.6) |  |
| Don't know | 12 (70.6) | 53 (37.6) | 65 (41.1) |  |
| No Response | 0 | 1 (0.7) | 1 (0.6) |  |
| **Disliked about TME** |  |  |  |  |
| Blood test | 2 (11.8) | 4 (2.8) | 6 (3.8) | 0.31 |
| Unable to go to work | 0 | 1 (0.7) | 1 (0.6) |  |
| Inadequate incentive | 0 | 1 (0.7) | 1 (0.6) |  |
| Other | 15 (88.2) | 135 (95.7) | 150 (94.9) |  |
| **If other, specify** |  |  |  |  |
| I like all | 8 (53.3) | 134 (99.3) | 142 (94.7) | **<0.001** |
| I did not participate | 7 (46.7) | 0 | 7 (4.7) |  |
| I did not like any | 0 | 1 (0.7) | 1 ((0.7) |  |
| **I think TME is important** |  |  |  |  |
| Yes | 8 (47.1) | 135 (95.7) | 143 (90.5) | **<0.001** |
| Don't know | 9 (52.9) | 6 (4.3) | 15 (9.5) |  |
| ***Yes,* because (n=143)** |  |  |  |  |
| It will make me/us healthy | 4 (50) | 81 (60) | 85 (59.4) | 0.54 |
| They are here to treat us | 1 (12.5) | 12 (8.9) | 13 (9.1) |  |
| We can eliminate malaria | 1 (12.5) | 12 (8.9) | 13 (9.1) |  |
| To treat/prevent malaria | 0 | 17 (12.6) | 17 (11.9) |  |
| Other | 2 (25) | 13 (9.6) | 15 (10.5) |  |
| **I would participate in future TME** | |  |  |  |
| Yes | 8 (47.1) | 130 (92.2) | 138 (87.3) | **<0.001** |
| No | 4 (23.5) | 3 (2.1) | 7 (4.4) |  |
| Yes, only if | 0 | 5 (3.5) | 5 (3.2) |  |
| Don’t know | 5 (29.4) | 3 (2.1) | 8 (5.1) |  |
| **Reason for current participation in TME*** | | |  |  |
| Because I wanted to get rid of malaria | 8 (80) | 109 (77.3) | 117 (77.5) | 0.6 |
| Because I wanted to be healthy | 5 (50) | 83 (58.9) | 88 (58.3) | 0.4 |
| Other | 0 | 2 (1.4) | 2 (1.3) | 0.87 |
| **I would not participate if not provided following items** | | |  |  |
| Free health care | 3 (30) | 65 (46.1) | 68 (45) | 0.62 |
| T shirts | 0 | 2 (1.4) | 2 (1.3) |  |
| All of the above | 2 (20) | 34 (24.1) | 36 (23.8) |  |
| Even if nothing | 5 (50) | 38 (27) | 43 (28.5) |  |
| Other | 0 | 2 (1.4) | 2 (1.3) |  |
| **I would recommend TME to others** | |  |  |  |
| Yes | 5 (41.7) | 38 (27) | 43 (28.1) | **0.004** |
| No | 4 (33.3) | 63 (44.7) | 67 (43.8) |  |
| Don’t Know | 2 (16.7) | 40 (28.4) | 42 (27.5) |  |
| No response | 1 (8.3) | 0 | 1 (0.7) |  |
| ***If Yes,* reasons** |  |  |  |  |
| Because it will keep us healthy | 3 (60) | 20 (52.6) | 23 (53.5) | 0.43 |
| I want to eliminate malaria | 1 (20) | 12 (31.6) | 13 (30.2) |  |
| I want to help them | 0 | 3 (7.9) | 3 (7) |  |
| I am scared that they can transmit me | 1 (20) | 1 (2.6) | 2 (4.7) |  |
| Some people do not understand | 0 | 2 (5.3) | 2 (4.7) |  |
| ***If No,* reasons** |  |  |  |  |
| I don’t know how to say | 3 (75) | 30 (47.6) | 33 (49.3) | 0.46 |
| Thy don't listen to me | 0 | 14 (22.2) | 14 (20.9) |  |
| I don’t want to talk | 1 (25) | 8 (12.7) | 9 (13.4) |  |
| Other | 0 | 11 (17.5) | 11 (100) |  |
| **Ways a village can help in the TME program** | | |  |  |
| I don’t know how to help | 5 (41.7) | 29 (20.6) | 34 (22.2) | 0.35 |
| I will help by participating in the project | 2 (16.7) | 45 (31.9) | 47 (30.7) |  |
| We all have to participate | 4 (33.3) | 58 (41.1) | 62 (40.5) |  |
| Other | 1 (8.3) | 9 (90) | 10 (6.5) |  |
| *Multiple answers were possible, percentage exceeds 100; analysis is based on a binary response "Yes" or "No" | | | | |

| **Table S5: Logistic regression on association between covariates with complete participation** | | | |  |  |  |
| --- | --- | --- | --- | --- | --- | --- |
|  | **Participation** |  | **Univariate Analysis** | **p-value** | **Multivariate analysis** | **p-value** |
|  | **Partial/none (n=17)** | **Complete (n=141)** | **Crude OR (95% CI)** |  | **AOR* (95% CI)** |  |
| **Covariates** | **Number (%)** | **Number (%)** |  |  |  |  |
| Sensitization by District health team/village health workers/study staffs | 13 (8.7) | 137 (91.3) | 10.53 (2.35 to 47.14) | **0.002** | 0.98 (0.04 to 20.54) | 0.99 |
| Attended meetings of TME | 11 (7.4) | 138 (92.6) | 25.09 (5.51 to 114.24) | **<0.001** | 12.01 (1.14 to 125.99) | **0.03** |
| Village head explained TME to you | 10 (7.5) | 124 (92.5) | 5.1 (1.71 to 15.19) | 0.003 | 4.54 (0.94 to 21.75) | 0.058 |
| Study staffs explained TME up to 30 times | 9 (6.4) | 132 (93.6) | 11.07 (3.65 to 33.61) | **<0.001** | 2.97 (0.58 to 15.19) | 0.19 |
| We get malaria from mosquito | 14 (9.2) | 139 (90.8) | 9.26 (1.21 to 70.63) | 0.032 | 0.12 (0.002 to 7.60) | 0.32 |
| Fever is the sign and symptoms of malaria | 8 (6.5) | 115 (93.5) | 4.97 (1.75 to 14.12) | **0.003** | 2.21 (0.46 to 10.63) | 0.32 |
| Malaria can be diagnosed through blood test | 10 (7.2) | 128 (92.8) | 6.89 (2.24 to 21.16) | **0.001** | 5.68 (1.00 to 32.30) | **0.05** |
| A healthy looking person can have malaria | 3 (4.8) | 60 (95.2) | 3.45 (0.95 to 12.56) | 0.06 | 0.97 (0.18 to 5.15) | 0.97 |
| Malaria can be eliminated by giving medicine to all the villagers | 6 (4.9) | 117 (95.1) | 8.93 (3.01 to 26.51) | **<0.001** | 3.87 (0.74 to 20.08) | 0.1 |
| Everyone from my house participated | 4 (4.7) | 81 (95.3) | 4.38 (1.36 to 14.12) | **0.013** | 4.27 (1.3 to 14.02) | **0.017** |
| Had complaints after round 1 | 1 (4) | 24 (96) | 3.28 (0.41 to 25.94) | 0.26 | 3.01 (0.33 to 26.97) | 0.32 |
| Had complaints with my HH members | 3 (7.7) | 36 (92.3) | 1.6 (0.43 to 5.88) | 0.48 | 0.89 (0.21 to 3.73) | 0.88 |
| Received enough information | 9 (6.2) | 137 (93.8) | 30.44 (7.68 to 120.62) | **<0.001** | 0.37 (0.01 to 11.89) | 0.58 |
| Medicine was given to kill malaria parasites | 8 (5.7) | 132 (94.3) | 16.5 (5.13 to 53.02) | **<0.001** | 6.77 (0.89 to 51.5) | 0.06 |
| Medicine did not cause many illnesses | 9 (7.4) | 113 (92.6) | 0.27 (0.09 to 0.78) | 0.016 | 1.16 (0.12 to 10.91) | 0.89 |
| Blood was taken to test for malaria parasite | 7 (5.5) | 121 (94.5) | 8.64 (2.94 to 25.33) | <0.001 | 0.76 (0.04 to 11.75) | 0.84 |
| I liked all about MDA | 8 (5.6) | 134 (94.4) | 21.53 (6.36 to 72.82) | <0.001 | 17.2 (1.66 to 177.99) | **0.017** |
| MDA is important | 8 (5.6) | 135 (94.4) | 25.31 (7.21 to 88.81) | **<0.001** | 14.94 (1.3 to 171.27) | **0.03** |
| I will participate if MDA happens next year | 8 (5.8) | 130 (94.2) | 13.29 (4.27 to 41.32) | <0.001 | 2.34 (0.27 to 20.05) | 0.43 |
| I would recommend MDA to others | 5 (11.6) | 38 (88.4) | 0.88 (0.29 to 2.68) | 0.83 | 0.18 (0.03 to 1.05) | 0.057 |

*(AOR=Adjusted Odds Ratio for age and sex)
